# A real-world pharmacovigilance analysis of omadacycline in FDA adverse event reporting system (FAERS) database

**DOI:** 10.3389/fphar.2025.1558868

**Published:** 2025-04-01

**Authors:** Xueping Shi, Chen Wang, Xiang Liu, Li Zou, Pei Guo

**Affiliations:** ^1^ College of Medical Technology, Chongqing Medical and Pharmaceutical College, Chongqing, China; ^2^ Department of Pharmacy, The First Affiliated Hospital of Chongqing Medical and Pharmaceutical College, Chongqing, China

**Keywords:** FAERS database, omadacycline, adverse events, pharmacovigilance analysis, antiinfective drug

## Abstract

**Objective:**

Omadacycline is a newly launched drug of tetracycline. Therefore, it is necessary to comprehensive evaluate reports on the safety of omadacycline in large, real-world populations. This study aimed to mine the adverse event (AE) signals related to omadacycline through the Food and Drug Administration Adverse Event Reporting System (FAERS) database.

**Methods:**

Data from the FAERS database from 1 January 2004 and 31 March 2024 were queried through OpenVigil 2.1. After the completion of data mapping, we collated and summarized key demographic and clinical characteristics of the reported cases. During the analysis, both Reporting odds ratio (ROR) and Bayesian confidence propagation neural network (BCPNN) were employed for the detection of AE signals.

**Results:**

We extracted a total of 452 suspected AE cases with omadacycline from the FAERS database. Among them, 69 positive signals were obtained using the ROR and BCPNN. The highest frequency reported systemic organ class (SOC) was gastrointestinal disorders. Common clinical AEs of omadacycline were detected in the FAERS database, such as nausea, vomiting, tongue discolouration, hepatic enzyme increased, and hypersensitivity. In addition, we identified potential unexpected serious AEs through disproportionality analysis, including eosinophilia, pancytopenia, internal haemorrhage, restless legs syndrome, hypoacusis, and tinnitus.

**Conclusion:**

In light of the growing use of omadacycline in clinical practice, routinely reviewing data from the FAERS database for signals of AE can help to ensure patient medication safety and enhance overall medical quality.

## Introduction

Tetracycline antibiotics are known for their broad-spectrum antibacterial activity, encompassing Gram-positive, most Gram-negative, anaerobic, and “atypical” (*Legionella pneumophila*, Chlamydophila pneumoniae, and *Mycoplasma* pneumoniae) bacterial species (LaPlante et al., 2022). However, the original clinical utility of the tetracyclines has been compromised due to the increasing prevalence of resistance among previously susceptible, common pathogens (Thaker et al., 2010). Therefore, obtaining semi-synthetic tetracycline through isolation or derivation has become a good way to improve drug resistance.

Omadacycline is a novel aminomethylcycline antibiotic, belonging to the third generation of tetracyclines. (Honeyman et al., 2015; Stets et al., 2019). Omadacycline exhibits structural distinctions from tetracycline, which include modifications at both the C7 position and the incorporation of an aminomethyl group at the C9 position (Draper et al., 2014). It can be reached high concentrations in lung by administered intravenously or orally. Omadacycline was approved by the US Food and Drug Administration (FDA) for the treatment of acute bacterial skin and skin structure infections, as well as community-acquired bacterial pneumonia in October 2018 (Burgos and Rodvold, 2019). Safety-related research of omadacycline has primarily been executed through clinical trials due to the short time of marketing, and the necessity of extended safety surveillance remains indispensable. The spontaneous reporting system database serves as the principal data repository for the detection of adverse event (AE) signals, and a number of rare adverse reactions have been identified through this method (Montastruc et al., 2016). This pharmacovigilance, retrospective study aims to survey the AEs related to omadacycline through FAERS database to provide valuable resource for future clinical use.

## Methods

### Data source

We performed a retrospective pharmacovigilance study in FAERS using OpenVigil 2.1 to evaluate the AE about omadacycline. OpenVigil is an advanced pharmacovigilance tool that is publicly accessible, enabling the extraction, cleaning, mining, and dissection of data from FAERS through the interface of open-FDA (Böhm et al., 2012). All AEs in the FAERS database are encoded using the preferred terms (PT) of the Medical Dictionary for Drug Regulatory Activities (MedDRA). The MedDRA is a unified standard terminology for recording and reporting adverse drug event data. There are five levels to the MedDRA, including lowest level terms (LLT), PT, high-level terms (HLT), high-level group terms (HLGT), and system organ class (SOC) (Brown, 2003).

### Data processing

Data collection for AEs related to omadacycline was conducted between 1 January 2004 with 31 March 2024. The AE reports were retrieved using the drug’s generic name and brand name, omadacycline and nuzyra respectively. During the creation of each AE report, reporters are required to categorize the drug’s role, designating them as primary suspect, secondary suspect, concomitant, or interacting. This study included primary and secondary suspect drugs that were responsible for AEs. Then we analyzed the data to extract the clinical characteristics of these reports. The clinical characteristics we collected include gender, age, patient outcomes, reported countries, and reported year.

We established specific inclusion and exclusion criteria. Inclusion criteria: (LaPlante et al., 2022): generic name is omadacycline, trade name is NUZYRA; (Thaker et al., 2010); Only reports where the role code was the primary and secondary suspect. Exclusion criteria: (LaPlante et al., 2022):AE reports not related to Omadacycline; (Thaker et al., 2010); reports where the role code was the concomitant and interacting.

### Signal mining

The method most often employed globally to detect the quantitative signals of AEs is known as the disproportionality analysis (Chuma et al., 2022). The algorithm for disproportionate analysis is detailed in Supplementary Table S1. In order to reduce the result bias caused by one method, this study uses the reporting odds ratio (ROR) method in the frequency method and the Bayesian confidence propagation neural network (BCPNN) method in the Bayesian method for signal detection. When both algorithms are positive, it is judged as a suspicious signal (Zhao et al., 2023).

## Results

### Clinical characteristics

**FIGURE 1:**
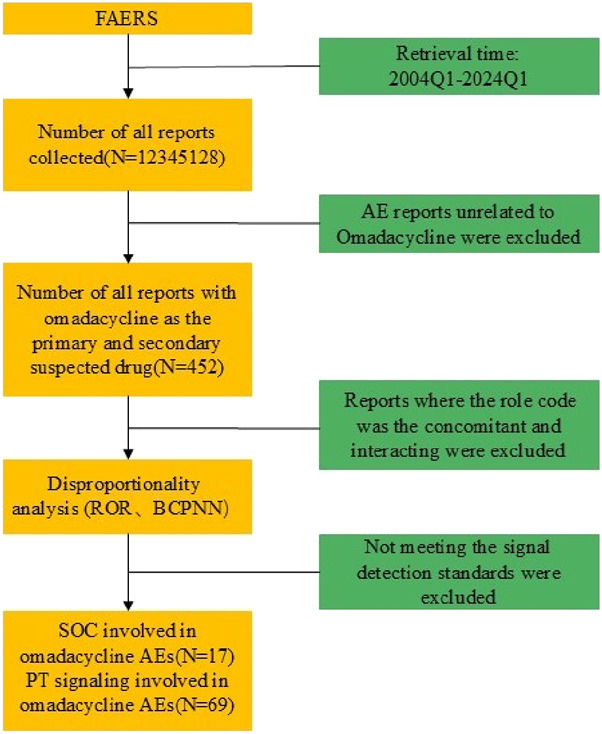
Flowchart for data filtering of omadacycline.

**FIGURE 2:**
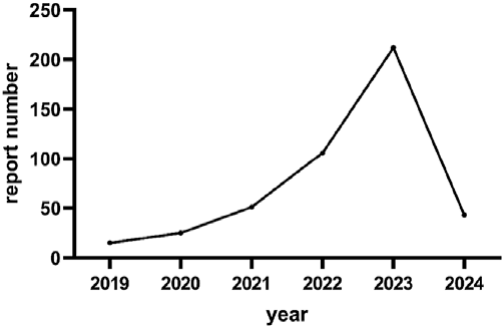
Number of reports with omadacycline in different years.

From 1 January 2004 to 31 March 2024, a total of 452 cases of omadacycline were found in the FAERS database. The specific flowchart of data filtering as shown in Figure 1. The Clinical characteristics of omadacycline-related AE reports were described in Table 1. Omadacycline-related AE reports were slightly higher in female patients (48.23%) than male patients (38.05%). Most reports focus on middle-aged (45–64 years old, 21.68%) people. In clinical outcome, hospitalization (initial or prolonged) was reported in 15.04%. The top one country reported was the United States (88.05%). An observation worth noting is the increasing trend observed with omadacycline-related AE reports year after year, culminating in a significant increase in 2023 as depicted in Figure 2.

**TABLE 1:** Clinical characteristics of omadacycline.

Characteristics	Number	Proportion
Gender		
Male	172	38.05%
Female	218	48.23%
Unkown	62	13.72%
Age (year)		
<18	6	1.33%
18–44	39	8.63%
45–64	98	21.68%
65–74	51	11.28%
≥75	47	10.40%
Unkown	211	46.68%
Outcome		
Death	36	7.96%
Disability	4	0.88%
Hospitalization-initial or prolonged	68	15.04%
Life-threatening	3	0.66%
Other	341	75.44%
Reported countries		
China	51	11.28%
Kuwait	1	0.22%
Republic of Serbia	1	0.22%
The United State	398	88.05%
Unkown	1	0.22%

### AE classifications at SOC level

**FIGURE 3:**
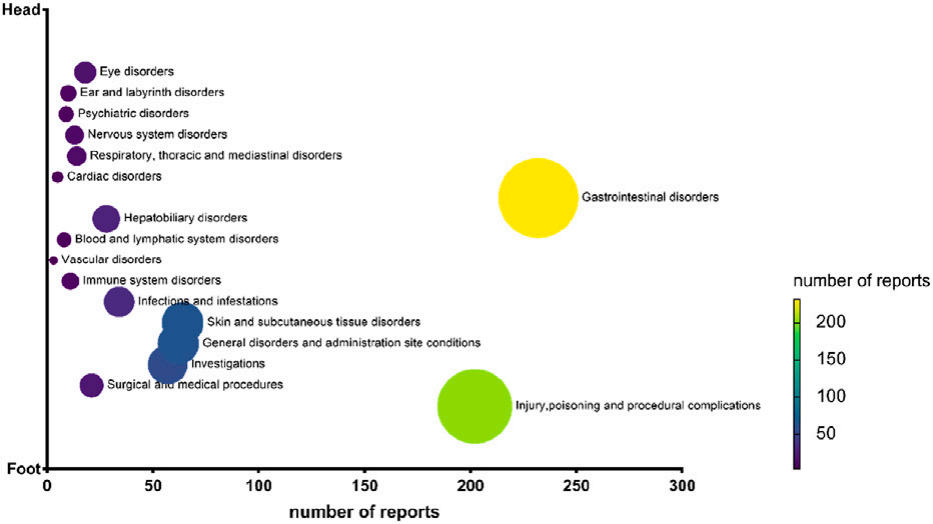
Number of reports for omadacycline related adverse events at the System Organ Class level.

We classified all Omadacycline-related AEs by SOC as shown in Figure 3. The results showed that the safety signal of omadacycline involved 17 SOCs. The most frequently reported SOC was gastrointestinal disorders (232, 29.33%), followed by injury, poisoning and procedural complications (202, 25.54%), skin and subcutaneous tissue disorders (64, 8.09%), General disorders and administration site conditions (62, 7.84%) and investigations (57, 7.21%).

### Disproportionality analysis at PT level

A total of 452 AE reports were analyzed, resulting in 69 safety signals which encompassed 17 SOCs (Table 2). The signals of the gastrointestinal disorders include nausea, vomiting, tooth discord and tongue discolouration. The Skin and subcutaneous tissue disorders is mainly manifested in rash, rash pruritic, rash erythematous. General disorders and administration site conditions include peripheral swelling, infusion site phlebitis, generalised oedema. The safety signals in investigations are hepatic enzyme increased, alanine aminotransferase increased, aspartate aminotransferase increased, blood pressure increased, blood alkaline phosphatase increased, blood bilirubin increased. In addition, some signals not mentioned on the label were detected, including vision blurred, eosinophilia, pancytopenia, internal haemorrhage, restless legs syndrome, hypoacusis and tinnitus.

**TABLE 2:** Signal strength of omadacycline at the PT level in FAERS database.

SOC/PT	N	ROR (95%CI)	IC (IC-2SD)
Gastrointestinal disorders			
Nausea*	81	6.32 (4.97,8.03)	2.34 (2.01)
Vomiting*	52	6.64 (4.97,8.87)	2.45 (2.05)
Diarrhoea*	33	3.00 (2.10,4.28)	1.43 (0.94)
Abdominal pain upper*	14	3.78 (2.22,6.44)	1.64 (0.97)
Abdominal discomfort*	12	3.73 (2.10,6.61)	1.60 (0.88)
Tooth discolouration*	12	233.13 (131.08,414.62)	3.62 (3.39)
Dry mouth	7	4.79 (2.27,10.10)	1.69 (0.89)
Gastrointestinal disorder	5	3.15 (1.31,7.61)	1.20 (0.27)
Lip swelling	5	7.76 (3.21,18.74)	1.86 (1.11)
Tongue discolouration	5	62.58 (25.90,151.25)	2.47 (2.12)
Trichoglossia	3	301.02 (96.12,942.67)	1.98 (1.72)
Vomiting projectile	3	61.68 (19.79,192.23)	1.93 (1.58)
Cardiac disorders			
tachycardia*	5	3.08 (1.27,7.43)	1.18 (0.24)
Blood and lymphatic system disorders			
Eosinophilia	4	13.56 (5.06,36.29)	1.94 (1.31)
Pancytopenia	4	4.17 (1.56,11.17)	1.35 (0.43)
Vascular disorders			
Internal haemorrhage	3	12.28 (3.94,38.23)	1.68 (1.02)
General disorders and administration site conditions			
Drug ineffective for unapproved indication	11	10.27 (5.64,18.68)	2.51 (1.90)
Drug resistance*	10	18.92 (10.11,35.42)	2.83 (2.30)
Unevaluable event	10	6.01 (3.21,11.26)	2.03 (1.32)
Disease progression	8	3.25 (1.62,6.54)	1.36 (0.54)
Peripheral swelling	7	2.81 (1.33,5.93)	1.18 (0.31)
Chest discomfort	5	2.83 (1.17,6.84)	1.11 (0.16)
Feeling hot	4	3.55 (1.33,9.51)	1.23 (0.28)
Infusion site phlebitis*	4	1224.66 (447.92,3348.38)	2.32 (1.98)
Generalised oedema	3	14.43 (4.63,44.92)	1.72 (1.11)
Skin and subcutaneous tissue disorders			
Rash*	25	3.06 (2.04,4.58)	1.45 (0.90)
Rash pruritic*	10	10.95 (5.85,20.50)	2.51 (1.89)
Rash erythematous	7	9.87 (4.68,20.82)	2.22 (1.54)
Skin discolouration	6	7.69 (3.44,17.22)	1.97 (1.23)
Skin mass	6	46.65 (20.83,104.46)	2.63 (2.23)
Rash papular	4	13.12 (4.90,35.12)	1.93 (1.29)
Drug eruption*	3	10.10 (3.24,31.44)	1.62 (0.91)
Skin lesion	3	6.15 (1.97,19.14)	1.42 (0.58)
Immune system disorders			
Hypersensitivity*	11	3.21 (1.76,5.83)	1.14 (0.67)
Psychiatric disorders			
Poor quality sleep*	9	24.72 (12.77,47.83)	2.86 (2.37)
Nervous system disorders			
Hypoaesthesia	7	2.50 (1.18,5.27)	1.06 (0.17)
Intercostal neuralgia	3	1472.87 (459.33,4722.85)	2.00 (1.59)
Restless legs syndrome	3	8.40 (2.70,26.14)	1.56 (0.80)
Respiratory, thoracic and mediastinal disorders			
Cough	10	2.07 (1.10,3.87)	0.90 (0.09)
Dysphonia	4	3.90 (1.46,10.43)	1.30 (0.37)
Surgical and medical procedures			
Hospitalisation	21	6.77 (4.37,10.50)	2.38 (1.82)
Injury, poisoning and procedural complications			
Off label use	133	11.43 (9.34,13.99)	2.98 (2.71)
Incorrect dose administered	37	9.28 (6.63,12.98)	2.84 (2.39)
Incorrect product administration duration	14	14.77 (8.67,25.15)	2.92 (2.38)
drug dose titration not performed	11	372.76 (204.13,680.68)	3.54 (3.32)
Wound secretion	4	64.60 (24.11,173.07)	2.23 (1.88)
Procedural pain*	3	6.29 (2.02,19.59)	1.43 (0.60)
Investigations			
Hepatic enzyme increased*	12	10.24 (5.77,18.18)	2.56 (1.95)
Alanine aminotransferase increased*	10	9.44 (5.05,17.68)	2.40 (1.76)
Aspartate aminotransferase increased*	10	11.14 (5.95,20.86)	2.52 (1.90)
Weight decreased	10	2.07 (1.10,3.87)	0.89 (0.09)
Blood pressure increased*	7	2.54 (1.21,5.37)	1.08 (0.19)
Blood alkaline phosphatase increased*	4	9.33 (3.49,24.98)	1.80 (1.08)
Blood bilirubin increased*	4	8.24 (3.08,22.07)	1.75 (0.99)
Infections and infestations			
Infection	7	2.79 (1.32,5.88)	1.17 (0.30)
Pathogen resistance	5	26.16 (10.83,63.19)	2.33 (1.83)
*Clostridium difficile* infection*	4	13.29 (4.96,35.56)	1.94 (1.30)
*Clostridium difficile* colitis*	3	18.40 (5.91,57.30)	1.78 (1.22)
Cutaneous sporotrichosis	3	3436.71 (1031.18,11453.82)	2.00 (1.43)
Lymphangitis	3	213.67 (68.35,667.95)	1.98 (1.72)
Mycobacterial infection	3	90.23 (28.94,281.36)	1.95 (1.64)
Osteomyelitis	3	9.39 (3.02,29.24)	1.60 (0.87)
Wound infection staphylococcal	3	114.39 (36.67,356.86)	1.96 (1.67)
Hepatobiliary disorders			
Hepatic function abnormal	28	44.82 (30.57,65.72)	4.12 (3.75)
Ear and labyrinth disorders			
Hypoacusis	5	4.51 (1.87,10.90)	1.50 (0.63)
Tinnitus	5	5.75 (2.38,13.89)	1.67 (0.86)
Eye disorders			
Vision blurred	10	3.82 (2.04,7.15)	1.58 (0.83)
Swelling of eyelid	5	52.51 (21.73,126.89)	2.45 (2.07)
Eyelid rash	3	187.45 (60.00,585.66)	1.98 (1.71)

Note: * means AEs, mentioned in the label.

## Discussion

Our study focused on the AEs of omadacycline and completed a comprehensive collection until recently to demonstrate the severity of AEs and possible connection with basic clinical features. To our knowledge, this study is the first pharmacovigilance study of AEs associated with omadacycline based on real-world data from the FAERS database.

Studies have shown that AEs of gastrointestinal are the most common AEs of omadacycline (O'Riordan et al., 2019a; O'Riordan et al., 2019b) (Overcash et al., 2019). These studies show that nausea and vomiting are the main gastrointestinal reactions, and the incidence rate of nausea and vomiting can reach 25.5% and 12.5% on the first and second days after drug use. Among the AEs detected in this study, AEs of gastrointestinal disorders were also the most common, including nausea, vomiting, diarrhoea, abdominal pain upper, tooth discolouration mentioned on the label, as well as dry mouth, lip swelling, tongue discolouration, trichoglossia and vomiting projectile not mentioned on the label. The findings from this study complement those of the preceding research, further bolstering the credibility of the findings. This suggests that when administering omacycline, clinicians should monitor patients for gastrointestinal adverse events, particularly during the initial 2 days of the treatment course. Furthermore, it has been observed that there is a strong correlation between Omadacycline and tooth discoloration [ROR = 233.13, 95% CI= (131.08, 414.62); IC = 3.62, IC-2SD = 3.39]. Similar to other tetracyclines, use during tooth development: late pregnancy, infancy, and childhood under the age of 8 years old, can lead to permanent tooth discoloration, a phenomenon that is more likely to occur with long-term use and also in frequent administration during shorter courses of treatment (Opal et al., 2019). Given these potential risks, it is not advisable to take Omacycline during tooth development.

Medications, as inducers of allergic reactions, are related to the severity of the reaction and may cause fatal allergic reactions (Jerschow et al., 2014). The Study have shown that fatal allergic reactions caused by medication account for 58.8% of allergy related deaths (Morikawa et al., 2016). Our study detected hypersensitivity reactions in the immune system disorders, as well as signals related to allergic reactions such as rash, rash pruritic, rash erythematous, rash papular, and drug eruption in the skin and subcutaneous tissue disorders. In clinical practice, it is important to carefully inquire about the history of allergies, select medications based on individual patient characteristics to reduce allergic reactions and related mortality rates.

It is worth noting that we also detected signals of Omadacycline related tachycardia in the Cardiac disorders. Non-clinical studies have demonstrated that omadacycline inhibits the binding of the neurotransmitter carbamylcholine to the M2 subtype of the muscarinic acetylcholine receptor, while remaining non-interactive with other muscarinic acetylcholine receptors or adrenergic receptors (Tanaka and Villano, 2016). This unique effect of omadacycline was not observed in previous *in vitro* studies with other tetracyclines. Although no other signals have been detected in the cardiac system, patients with potential heart disease or heart risk factors should still be alert to AEs related to the cardiac system.

There is a significant association between the administration of omadacycline and hepatic AEs, such as hepatic function abnormal, hepatic enzyme increased, alanine aminotransferase increased, aspartate aminotransferase increasedblood, alkaline phosphatase increased, and blood bilirubin increased. Data from a Phase I clinical trial indicate that intravenous administration of omadacycline at doses ≥300 mg may lead to transient increases in serum alanine aminotransferase (ALT) levels. Furthermore, dose-limiting changes were observed at an oral dose of 600 mg (Bundrant et al., 2018). Patients who use tetracycline often experience alterations in liver function tests, with particularly ascend of liver aminotransferases (Moseley et al., 2013). Therefore, hepatic AEs may be common AEs among tetracyclines. Hepatotoxicity is a significant contributor to treatment discontinuation, necessitating regular liver function monitoring. In cases of severe hepatic dysfunction, it is crucial that medication be promptly reduced or ceased.

The rational use of antibiotics is the key to improving efficacy, reducing the incidence of AEs, and delaying the occurrence of bacterial resistance. This study detected 10 cases of omadacycline related drug resistance, and its resistance risk deserves further attention. In addition, other signals include vision blurred, eosinophilia, pancytopenia, internal haemorrhage, restless legs syndrome, and hypoacusis and tinnitus, although this is not specifically mentioned on the label, and there have been no reports of adverse events caused by omadacycline. These AEs may be false positive data or clinically rare or unrecognized ADE signals. Therefore, these AEs that are not explicitly included in the instruction manual warrant further attention and validation in clinical practice.

Through the utilization of a spontaneous reporting system and data mining applied to signal detection, numerous instances of potential or delayed AEs can be identified. This study detected safety signals in the gastrointestinal, cardiac, hepatobiliary, skin systems and so on, with a particular focus on tooth discolouration, tachycardia, hepatic function abnormal, hypersensitivity, and some AEs not mentioned on the label, including vision blurred, eosinophilia, pancytopenia, restless legs syndrome, hypoacusis, and tinnitus. However, this study also has some limitations. The safety signals identified in the present study are indicative of a statistical correlation between omadacycline and the AEs, but they do not conclusively establish a causal relationship between the drug and the AE. Additional rigorous clinical trials are required to further investigate this potential causal association. Secondly, given the limited number of reports on most unexpected AEs, clinical settings should enhance surveillance of these AEs. In addition, since the data in the FAERS database does not include the total number of drug users. Consequently, only the magnitude of the association between these drugs and AEs can be accurately quantified, while the precise incidence of such events remains undetermined. Finally, the database has inherent limitations of being a spontaneous reporting database, such as the inability to obtain information on patients’ medical history, severity of illness, and other related information, as well as incomplete information on dosage, route of administration, and duration of medication (Zhou et al., 2023).

## Conclusion

This study was based on the FAERS database and used disproportionate analysis to mine and analyze the AEs of omadacycline. The common AEs (nausea, vomiting, tachycardia, hepatic enzyme increased) are consistent with the label. Furthermore, our research had identified several unexpected AE signals, such as eosinophilia, pancytopenia, internal haemorrhage, restless legs syndrome, hypoacusis, and tinnitus, which warrant clinical consideration. This study is beneficial for compensating for the scarcity of clinical samples, while also identifying rare AEs to omadacycline. The findings of this study have the potential to provide valuable insights for informed clinical medication decisions. Nevertheless, given the limitations inherent to the FAERS database, further studies are required to fully elucidate the causal relationship between these AEs and omadacycline.

## Data Availability

The original contributions presented in the study are included in the article/Supplementary Material, further inquiries can be directed to the corresponding authors.
