# Supplementary material for: A real-world pharmacovigilance analysis of omadacycline in FDA adverse event reporting system (FAERS) database
Source: Front Pharmacol. 2025 Apr 1;16:1558868. doi: 10.3389/fphar.2025.1558868 (PMC11996809; doi:10.3389/fphar.2025.1558868)
Supplement: Supplementary file 1 [file Table1.docx]

Disproportionality analysis algorithm

| Item | Target AEs | Other AEs | Sums |
| --- | --- | --- | --- |
| Target drug | a | b | a+b |
| Other drugs | c | d | c+d |
| Sums | a+c | b+d | a+b+c+d |
